# Progress in Cancer

**Published:** 1905-07-15

**Authors:** 


### PROGRESS IN CANCER.

**Endothelioma of Pleura.**—Under this title Osler and Thayer[1] describe cases of malignant disease of the pleura as noted in German literature. The appearance of the growth is either that of a thick fibroid pleura or, more commonly, it has small cartilaginoid nodules scattered over the surface. Microscopically the growth consists of an interlacing network of tubules lined by low epitheloid cells. The symptoms are those of a chronic progressive pleurisy, the effusion becoming hæmorrhagic after one or two tappings. A suggestive sign is the drawing in of the intercostal spaces over the growth. All reported cases have been in males, and usually on the left side.

**Primary Carcinoma of the Duodenum.**—Brill[2] reviews the literature of recorded cases of primary cancer of the duodenum. Of all cases of malignant disease of the intestine, those of the small gut only form 2·3 per cent. Of the various parts of the small intestine the jejunum is never attacked, and the duodenum rather more frequently than the ileum, if cases starting in the ileo-cæcal valve be excluded. Of the three parts of the duodenum, the second is much the most frequently attacked, and the third the most seldom. The growth is generally composed of columnar cells, but spheroidal-celled growths occur rarely. Both these are often subject to necrotic or colloid change. They tend to assume an annular form, producing marked stricture of the lumen. The patients are generally men (three to one) of an average age of 55, and the duration of life about seven months. The symptoms differ according to the site of the growth. If above the biliary papilla, only signs similar to those of pyloric obstruction will be present. If involving the bile duct, then chronic and persistent jaundice is added; and lastly, in the rare cases where the growth is below, and does not involve the papilla of Vater, there is a recurrent vomiting of bile and pancreatic fluid. In the majority of cases only palliative treatment is possible, but where the disease is limited to the first part of the duodenum an excision should be feasible.

**A New Theory as to the Cause of Malignant Disease.**—The *Medical Record*[3] reviews Kelling's recent theory of the origin of malignant growths from the embryonal cells taken into the digestive canal in animal food. Triturations of chick embryos injected into dogs gave rise to malignant growth. A portion of macerated human gastric carcinoma was injected into a rabbit, and the serum of the latter gave a precipitin reaction with not only the tumour extract, but also with chicken albumen. Also, the serum of patients suffering from cancer in the great majority of cases gave a positive precipitin reaction with either chicken or pig albumen. One case of early diagnosis of a cancer of the stomach was made by this reaction.

**Remarkable "Dermoid" Cyst of Ovary.**—Shattuck[4] showed at the Pathological Society in London a dermoid cyst of the ovary into which a well-developed teratoma (or ill-developed fœtus) projected. It consisted of the lower half of a fœtal body, showing both lower limbs, pelvic girdle, vertebral column, spinal cord, containing medullated nerves, a vulva, pubic hair, and a short coil of intestine. He propounds a theory which he terms "epigenesis" to account for ovarian and testicular dermoids. According to this theory, the embryonic ova are fertilised by spermatozoa which have been included in the fœtus; and, as the result of this, teratomata arise in the genital gland. In the case of the testis it is necessary to assume that a primitive hermaphrodite condition has obtained, and that a few ova have developed in the testis, and then been fertilised by the spermatozoa in the gland.

**The Treatment of Cancer.**—In the Bradshaw lecture Mayo Robson[5] lays stress on the infectivity of cancer. Cancer can easily be inoculated from one white mouse to another, the transferred cells forming a parasitic tumour. And even without inoculation healthy white mice are reported to have become cancerous by mere contact with infected mice. Also fleas taken from cancerous mice rapidly infect healthy mice. Lastly, many cases are on record of a man developing cancer of the penis whose wife had cancer of the uterus. That it is auto-infective is still more abundantly clear from the many cases of infection of the operation wound by cancer particles, leading to a recurrence in the scar, and by the formation of a cancerous ulcer on any skin surface which comes in contact with another similar ulcer—*e.g.*, an ulcerated cancer of the breast infected the skin on the inner side of the arm. These facts so strongly suggest that cancer is contagious that it becomes imperative to guard against the spread of the disease by burning or disinfecting all articles contaminated by cancer discharges. In the surgical prevention of cancer, the first possibility is the avoidance of irritation of skin and mucous membrane—*e.g.*, by rough pipes causing epithelioma of the lip, soot causing sweep's cancer, phimosis—epithelioma of the penis. The second demand is to remove pre-cancerous conditions before malignant disease has developed. Such are chronic ulcers and warts of the lips, tongue, and cheek; leukoplakia; moles and warts showing signs of ulceration or enlargement; cholelithiasis, with ulceration of the bile-ducts; in the breast, eczema of the nipple, chronic interstitial mastitis, cysts, adenomata, and indurations following injury. In the stomach chronic ulcers are the precursors of many cases of cancer, and this occurs especially in ulcers near the pylorus. The persistence of hyperchlorhydria, with symptoms of malignant disease, makes the origin of any particular case of cancer from a pre-existing ulcer probable. In the uterus cancer is often preceded by the scars of laceration, and sometimes by simple myomata, each of which can readily be cured. As regards the result of radical treatment, the author quotes statistics from the most eminent specialists in each department, with the following result, as judged by survival beyond the three years' limit:—Cancer of the breast, 50 per cent.; cancer of the stomach, 9 per cent.; of the tongue, 20 per cent.; intrinsic cancer of larynx, 85 per cent.; of the colon, 36 per cent.; of the rectum, 17 per cent.; of the gall bladder and liver, 40 per cent.; of the lip, 53 per cent.; of the penis, 33 per cent.; of the uterus, 35 per cent. In considering palliative operations great value is attached to gastrostomy for disease of the œsophagus, gastro-enterostomy for diseases of the stomach, and short-circuiting operations for cancer of the intestine.

1 Amer. Jour. of Med. Sci., October 1904. 2 Ibid., November 1904. 3 Med. Rec., Nov. 19, 1904. 4 Lancet, Nov. 5, 1904. 5 Lancet, Dec. 3, 1904.

